# Short-term preliminary clinical & functional results by TRD stage from HIPOCRAT-ESK study: a cohort, non-interventional, prospective, real-world study of functional remission in TRD patients treated with Esketamina nasal spray

**DOI:** 10.1192/j.eurpsy.2026.11435

**Published:** 2026-07-31

**Authors:** T. Caraballo Lopez, M. García Dorado, N. Cardoner Álvarez, Á. Ibáñez Cuadrado, S. Benavente López, P. Alonso Ortega

**Affiliations:** 1 Johnson & Johnson, Madrid; 2 Hospital Santa Creu i Sant Pau, Barcelona; 3 Hospital Ramón y Cajal; 4 Hospital Infanta Elena, Madrid; 5 Hospital Bellvitge, Barcelona, Spain

## Abstract

**Introduction:**

Treatment-resistant depression (TRD) patients experience a higher burden of illness and worse clinical prognosis. The potential for functional improvement decreases with symptom chronicity and treatment failure.

**Objectives:**

To report the initial functional results of HIPOCRAT-ESK aims to assess functional remission rates in TRD patients under ESK NS treatment at different TRD stages, evaluating if early intervention relates to more beneficial functional and clinical outcomes in Spanish clinical practice.

**Methods:**

HIPOCRAT-ESK is a cohort, non-interventional, prospective, real-world study evaluating functions remission as a primary endpoint in TRD patients treated with ESK NS as per the Spanish clinical practice. It aims to include a total sample of 300 TRD patients and complete a prospective follow-up of 12 months. The study will explore the impact of TRD stage (TRD4, TRD5 or TRD6+, depending on the number of prior failed antidepressants) upon functional and clinical response and remission rates. We present the short-term clinical and functional results by TRD stage.

**Results:**

Clinical evolution results show that an earlier use of ESK NS correlates with higher MADRS response and remission rates (Table 1). Functional remission rates also were higher in those patients with a lesser number of prior failed antidepressant therapies (TRD4 and TRD% cohorts) in comparison with more chronic patients (TRD6+ cohort) (Table 2). PROs (PHQ-9 and EQ-5D-5L) results also pointed to a more beneficial clinical and impact perception from the patients when ESK NS was introduced as earlier treatment lines. In the case of PHQ-9 is especially relevant the amelioration of Pain/discomfort and anxiety/depression items in patients in the earlier cohorts (TRD4).

**Table 1.** MADRS score evolution by TRD Stage Table 1. Long description.

POPULATION	ITT		TRD4		TRD5		TRD6+	
INDUCTION PHASE DAY	Day 15	Day 28	Day 15	Day 28	Day 15	Day 28	Day 15	Day 28
**Clinical reponse n (%)**	12 (15.58%)	15 (21.43%)	8 (23.53%)	8 (27.59%)	3 (14.29%)	5 (25%)	0,00	2 (9.52%)
**Clinical Remission n (%)**	0,00	5 (7.14%)	0,00	3 (10.34%)	0,00	1 (5%)	0,00	1 (4.76%)

**Table 2.** SDS score evolution by TRD Stage Table 2. Long description.

POPULATION	ITT		TRD4		TRD5		TRD6+	
INDUCTION PHASE DAY	Day 15	Day 28	Day 15	Day 28	Day 15	Day 28	Day 15	Day 28
** Functional remission n (%)**	3 (3.9%)	7 (10%)	1 (2.94%)	2 (6.90%)	2 (9.52%)	4 (20%)	0,00	1 (4.76%)

**Conclusions:**

This analysis shows that early optimization of treatment leads to better results in the short term and improves the probability of achieving complete functional recovery. The novel mechanism of action of ESK NS, focused on restoring synaptic plasticity and connectivity between key brain regions, could explain the clinical and functional results observed in this study.

**Disclosure of Interest:**

T. Caraballo Lopez: None Declared, M. García Dorado Employee of: Johnson & Johnson Full-time empolyee, N. Cardoner Álvarez Grant / Research support from: Has recieved research support from Johnson & Johson, Consultant of: Has participated as consultant for Johnson & Johnson, Á. Ibáñez Cuadrado Grant / Research support from: Has recieved research support from Johnson & Johnson, Consultant of: Has participated as consultant for Johnson & Johnson, S. Benavente López Grant / Research support from: Has recieved research support from Johnson & Johnson, Consultant of: Has participated as consultant for Johnson & Johnson, P. Alonso Ortega Grant / Research support from: Has recieved Research support from Johnson & Johnson

## Long descriptions

### Table 1. Long description

The table is organized into nine columns. The first column lists the clinical metrics, while the subsequent columns are grouped by population: I T T, T R D 4, T R D 5, and T R D 6 plus. Each population is subdivided into two time points: Day 15 and Day 28 of the induction phase.

* Clinical response n (percentage):

- I T T: 12 (15.58 percent) at Day 15; 15 (21.43 percent) at Day 28.

- T R D 4: 8 (23.53 percent) at Day 15; 8 (27.59 percent) at Day 28.

- T R D 5: 3 (14.29 percent) at Day 15; 5 (25 percent) at Day 28.

- T R D 6 plus: 0.00 at Day 15; 2 (9.52 percent) at Day 28.

* Clinical Remission n (percentage):

- I T T: 0.00 at Day 15; 5 (7.14 percent) at Day 28.

- T R D 4: 0.00 at Day 15; 3 (10.34 percent) at Day 28.

- T R D 5: 0.00 at Day 15; 1 (5 percent) at Day 28.

- T R D 6 plus: 0.00 at Day 15; 1 (4.76 percent) at Day 28.

Navigate back to Table 1..

### Table 2. Long description

The table is organized into two header rows and one data row.

Header Row 1 (Population):

- I T T

- T R D 4

- T R D 5

- T R D 6 plus

Header Row 2 (Induction Phase Day): Each population is split into Day 15 and Day 28 columns.

Data Row (Functional remission n percentage):

- I T T: 3 (3.9 percent) at Day 15 and 7 (10 percent) at Day 28.

- T R D 4: 1 (2.94 percent) at Day 15 and 2 (6.90 percent) at Day 28.

- T R D 5: 2 (9.52 percent) at Day 15 and 4 (20 percent) at Day 28.

- T R D 6 plus: 0,00 at Day 15 and 1 (4.76 percent) at Day 28.

Navigate back to Table 2..

